# Crystal structure of a trigonal polymorph of aqua­dioxidobis(pentane-2,4-dionato-κ^2^ *O*,*O*′)uranium(VI)

**DOI:** 10.1107/S2056989021011063

**Published:** 2022-01-01

**Authors:** Alejandro Hernandez, Indranil Chakraborty, Gabriela Ortega, Christopher J. Dares

**Affiliations:** a Department of Chemistry and Biochemistry, Florida International University, 11200 SW 8th St., Miami, FL 33199, USA

**Keywords:** crystal structure, uranium complex, dioxo species, X-ray structure, hydrogen bonding

## Abstract

The title compound, [UO_2_(acac)_2_(H_2_O)] consists of a uran­yl(VI) unit ([O=U=O]^2+^) coordinated to two monoanionic acetyl­acetonate (acac, C_5_H_7_O_2_) ligands and one water mol­ecule. The asymmetric unit includes a one-half of a uranium atom, one oxido ion, one-half of a water mol­ecule and one acac ligand. The coordination about the uranium atom is distorted penta­gonal–bipyramidal. The acac ligands and O_w_ atom comprise the equatorial plane, while the uranyl O atoms occupy the axial positions. Inter­molecular hydrogen bonding between complexes results in the formation of two-dimensional hexa­gonal void channels along the *c-*axis direction with a diameter of 6.7 Å. The monoclinic (*P*2_1_/*c* space group) polymorph was reported by Alcock & Flanders [(1987). *Acta Cryst.* C**43**, 1480–1483].

## Abstract 2

[UO_2_(acac)_2_(H_2_O)] is constructed from one uran­yl(VI) unit, two monoanionic acetyl­acetonate (acac) ligands and one aqua ligand. The U atom exhibits a UO_7_ distorted penta­gonal–bipyramidal coordination geometry; four O atoms from two chelating bidentate acac ligands and one O atom of a aquo ligand (O_w_) form the equatorial plane while two uran­yl(VI) O atoms are located at the axial positions.

## Chemical context

Nuclear forensics applications often require the development of source materials for isotope-dilution mass-spectrometry measurements. One method for preparing actinide source materials includes the preparation of volatile compounds which can be deposited onto a conductive surface from the vapor phase. An alternative method involves electrochemical reduction to the zero-valent metal with concurrent deposition onto the electrode surface. This requires an organo-soluble actinide precursor. Hexavalent actinide complexes with β-diketonates are possibilities for either of these methods. They are neutrally charged, and with appropriate substituents on the β-diketonate may be volatile (Johnson *et al.*, 2017 ▸). β-Diketonates also provide a platform to prepare the organosoluble precursors required for electrochemical reduction. 
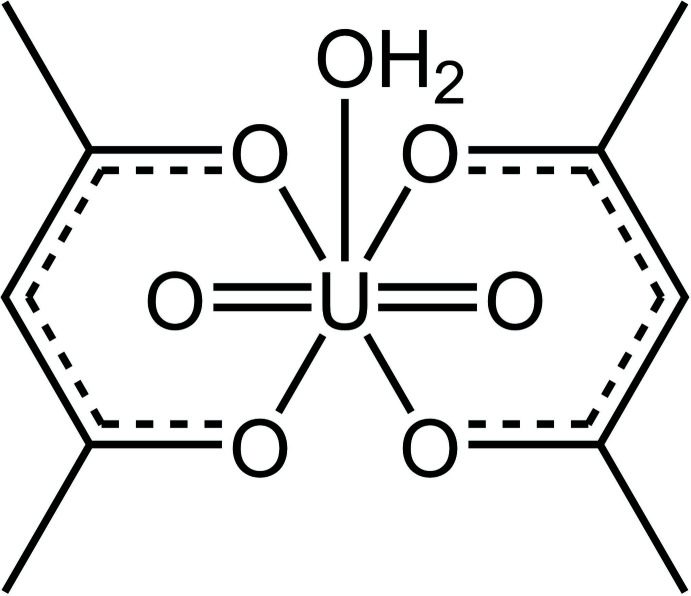


## Structural commentary

**Figure 1:**
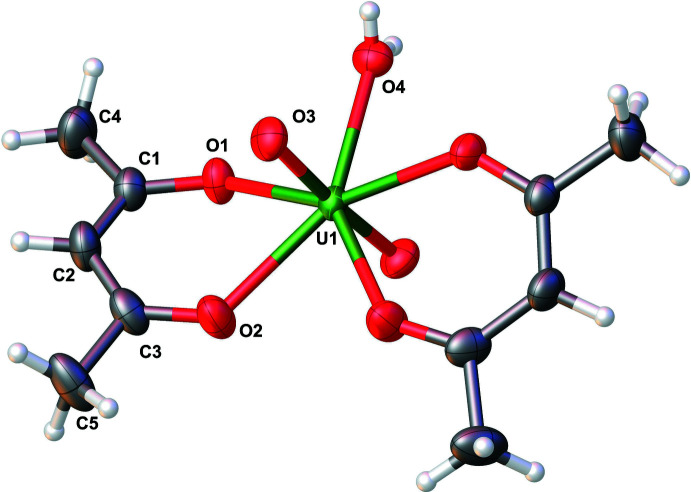
Mol­ecular structure of aqua­dioxidobis(pentane-2,4-dionato-κ^2^ *O*,*O*′)uranium(VI). Displacement ellipsoids are drawn at the 50% probability level.

The mol­ecular structure of the title compound, [UO_2_(acac)_2_(H_2_O)] **1**, was determined by single crystal X-ray diffraction. An *ORTEP* plot of the mol­ecular structure is shown in Fig. 1 ▸ and selected geometric parameters are listed in Table 1 ▸. The complex crystallizes in the trigonal *P* 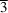 c1 space group with one-half mol­ecule per asymmetric unit, while the other half is generated by a twofold axis running through the U and O_w_ atoms. In the mol­ecular structure, the U^VI^ center resides in a distorted penta­gonal–bipyramidal coordination environment, with the equatorial positions occupied by the four O atoms of two chelating monoanionic acac ligands, and one water mol­ecule, while the two oxido ions reside at the axial positions *trans* to each other. The equatorial plane composed of O1, O2 (and the two other symmetry-equivalent atoms) and O4 deviates noticeably from planarity [with a mean deviation of 0.172 (3) Å]. The two six-membered chelate rings composed of U1, O1, C1, C2, C3 and the symmetry-equivalent atoms deviate significantly from planarity [mean deviation, 0.211 (3) Å]. The dihedral angle between the two chelate best-fit planes is 26.02 (13)°.

## Supra­molecular features

**Figure 2:**
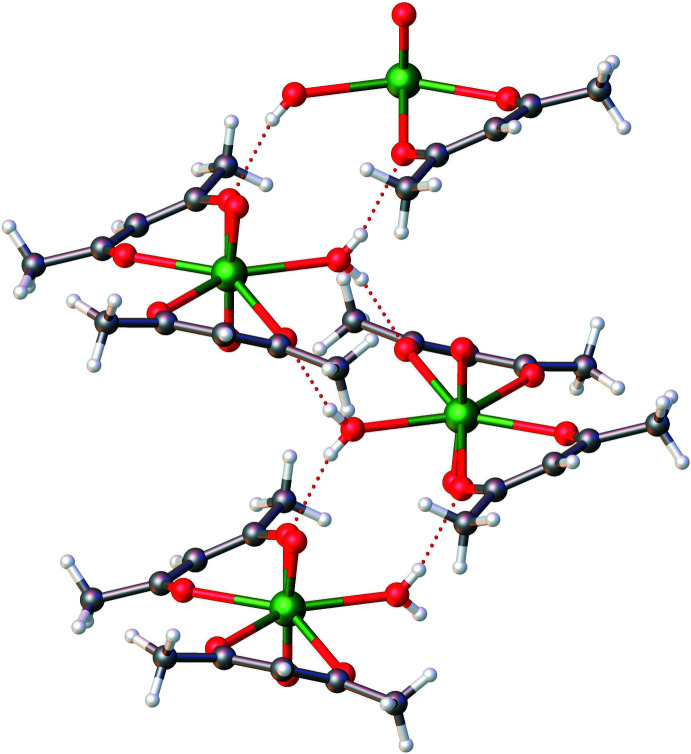
Representation of the O—H⋯O inter­molecular hydrogen-bonding inter­actions in aqua­dioxidobis(pentane-2,4-dionato-κ^2^ *O*,*O*′)uranium(VI)

**Figure 3:**
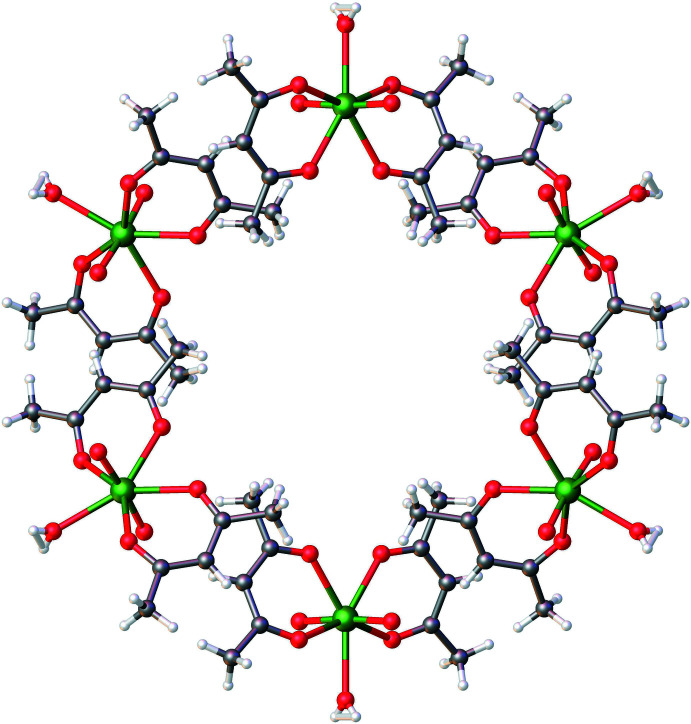
Formation of a ring structure extracted from the packing pattern of aqua­dioxidobis(pentane-2,4-dionato-κ^2^ *O*,*O*′)uranium(VI).

Examination of the extended structure revealed a prominent inter­molecular hydrogen bonding inter­action (O4—H4⋯O1) involving one of the O atoms of the acac ligand and the O_w_ atom (Fig. 2 ▸, Table 2 ▸). This inter­action results in pairing of two mononuclear units, eventually consolidating the extended structure. The packing pattern along the *c*-axis direction reveals an extended pattern with considerably large hexa­gonal void channels, each one surrounded by six other smaller void channels. The void volume was determined using contact surface maps (which offer an estimate of the volume that could be filed by guest mol­ecules) to be 325.41 Å^3^, representing 13.4% of the unit-cell volume (Barbour, 2006 ▸). This inter­esting packing pattern is shown in Figs. 3 ▸ and 4 ▸.

## Database survey

A scrutiny of the CSD (*Conquest* version 2.0, 2021; Groom *et al.*, 2016 ▸) revealed two other crystal structures of [UO_2_(acac)_2_(H_2_O)], **2** and **3**, available for comparison [there are few others for which coordinates are not available, see: Dornberger-Schiff & Titze (1969 ▸) and Comyns *et al.* (1958 ▸)]. Structure **2** is a polymorph of **1**, while structure **3** is a pyrazine solvate of [UO_2_(acac)_2_(H_2_O)]. Selected metric parameters of **1**–**3** are listed in Table 3 ▸. Structures **1** and **2** differ in their crystal packing arrangement. Whereas **1** crystallizes in the trigonal *P* 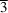 c1 space group with half a mol­ecule per asymmetric unit, **2** crystallizes in the monoclinic *P*2_1_/*c* space group with the asymmetric unit consisting of the complete mol­ecule (Alcock & Flanders, 1987 ▸). The differences in metric parameters between structures **1** and **2** are subtle (albeit with a slightly longer U—O_w_ distance in **2**), the differences being attributed to the different crystal packing. In case of **1**, classical hydrogen bonding dictates the packing pattern, while in **2**, the inter­molecular inter­actions are chemically inconsequential. Structure **3** is also quite similar to **1** and **2**. However, **3** crystallizes in the triclinic *P* 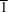 space group with one inter­stitial pyrazine mol­ecule (the asymmetric unit contains the full [UO_2_(acac)_2_(H_2_O)] mol­ecule and two half-mol­ecules of pyrazine). The pyrazine mol­ecules within the structure are engaged in hydrogen bonding with the coordinated water mol­ecule [O_w_—H—-N(pyrazine), H⋯N = 1.95 (2) Å and O_w_⋯N = 2.765 (5) Å; Kawasaki & Kitazawa 2008 ▸], thus preventing the supra­molecular organization of the uranium complex (seen in **1**). Inter­estingly, the lower density of **1** compared to those of **2** and **3** (1.99, 2.27 and 2.03 g cm^−3^ for **1**, **2**, and **3** respectively) is attributed to the large voids within the hexa­gonal channels.

## Synthesis and crystallization

To a vial containing 377 mg (3.8 mmol) of 2,4-penta­nedione (acac) in 7 mL of THF was added 20 mL of an aqueous solution containing 0.94 mmol of UO_2_(OAc)_2_(H_2_O)_2_. The reaction mixture rapidly changed color from colorless to yellow. A 10 *M* aqueous solution of KOH was added dropwise to the reaction mixture until the pH was approximately 9 (500 µL). The color of the solution intensified to a dark yellow concurrent with the formation of a suspension. The suspension was extracted with 50 mL of toluene, and the resultant yellow organic solution was dried over Na_2_SO_4_. After reducing the volume by 50% at reduced pressure, the remaining solution was allowed to evaporate at room temperature. Over the course of 1 week, yellow crystals were formed (150 mg, 34% yield).

## Refinement

Crystal data, data collection and structure refinement details are summarized in Table 4 ▸. The water O atom was freely refined. C-bound H atoms were positioned geometrically (C—H = 0.03–0.96) and refined as riding with *U*_iso_(H) = 1.2–1.5*U*_eq_(C).

## Supplementary Material

Crystal structure: contains datablock(s) I. DOI: 10.1107/S2056989021011063/ex2049sup1.cif

Structure factors: contains datablock(s) I. DOI: 10.1107/S2056989021011063/ex2049Isup2.hkl

CCDC reference: 2117155

Additional supporting information:  crystallographic information; 3D view; checkCIF report

## Figures and Tables

**Figure 4:**
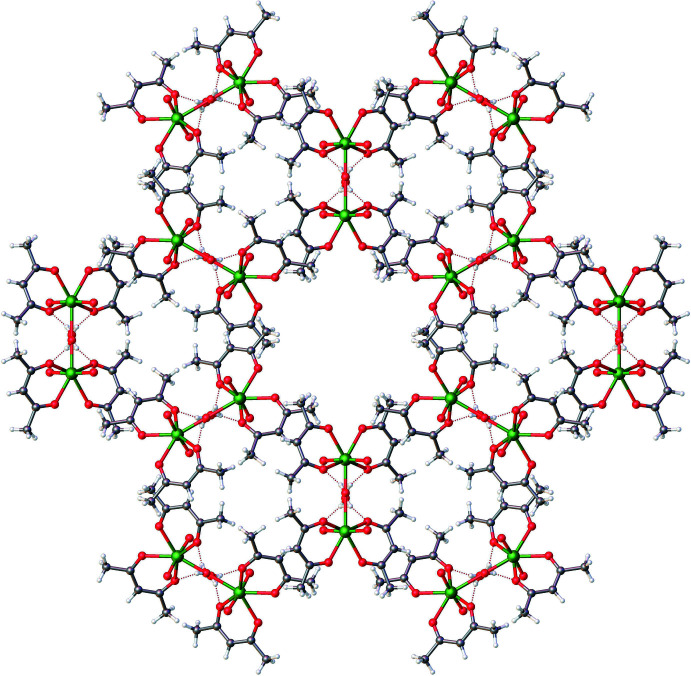
Packing pattern of aqua­dioxidobis(pentane-2,4-dionato-κ^2^ *O*,*O*′)uranium(VI) along the *c* axis.

**Table 1:** Selected geometric parameters (Å, °)

U1—O4	2.392 (4)	U1—O2	2.361 (3)
U1—O1	2.381 (2)	U1—O3	1.769 (2)
			
O1—U1—O4	75.17 (6)	O3—U1—O1^i^	86.27 (10)
O1^i^—U1—O1	150.33 (12)	O3—U1—O1	92.50 (11)
O2—U1—O4	144.24 (7)	O3—U1—O2	86.10 (11)
O2^i^—U1—O1	139.25 (9)	O3^i^—U1—O2	97.81 (12)
O2—U1—O1	70.00 (9)	O3—U1—O3^i^	175.21 (17)
O3—U1—O4	87.61 (9)		

Symmetry code: (i) x-y, -y, -z+{\script{3\over 2}}.

**Table 2:** Hydrogen-bond geometry (Å, °)

*D*—H⋯*A*	*D*—H	H⋯*A*	*D*⋯*A*	*D*—H⋯*A*
O4—H4⋯O1^ii^	0.72 (6)	2.01 (5)	2.704 (3)	164 (7)

Symmetry code: (ii) -x+1, -y, -z+1.

**Table 3:** Comparison of selected metric parameters (Å)

	**1** *^*a*^*	**2** *^*b*^*	**3** *^*c*^*
U—O(H_2_O)	2.392 (4)	2.489 (8)	2.409 (4)
U=(oxo)	1.769 (2)	1.743 (6)	1.776 (3)
U—O(acac)	2.371 (3)	2.339 (6)	2.354 (4)

Notes: (*a*) this work; (*b*) Alcock & Flanders (1987 ▸); (*c*) Kawasaki & Kitazawa (2008 ▸).

**Table 4:** Experimental details

Crystal data	
Chemical formula	[UO_2_(C_5_H_7_O_2_)_2_(H_2_O)]
*M*_r_	486.26
Crystal system, space group	Trigonal, *P*\overline{3}*c*1
Temperature (K)	298
*a*, *c* (Å)	19.5774 (9), 7.3264 (5)
*V* (Å^3^)	2431.8 (3)
*Z*	6
Radiation type	Mo *K*α
μ (mm^−1^)	10.03
Crystal size (mm)	0.30 × 0.15 × 0.05

Data collection	
Diffractometer	Bruker D8 Quest PHOTON II
Absorption correction	Multi-scan (*SADABS*; Krause *et al.*, 2015 ▸)
*T*_min_, *T*_max_	0.457, 0.745
No. of measured, independent and observed [*I* > 2σ(*I*)] reflections	26571, 1493, 1426
*R*_int_	0.027
(sin θ/λ)_max_ (Å^−1^)	0.603

Refinement	
*R*[*F* ^2^ > 2σ(*F* ^2^)], *wR*(*F* ^2^), *S*	0.016, 0.042, 1.13
No. of reflections	1493
No. of parameters	89
H-atom treatment	H atoms treated by a mixture of independent and constrained refinement
Δρ_max_, Δρ_min_ (e Å^−3^)	0.51, −1.06

Computer programs: *APEX3* and *SAINT* (Bruker, 2016 ▸), *SHELXT2018/2* (Sheldrick, 2015*a* ▸), *SHELXL2018/3* (Sheldrick, 2015*b* ▸), and *OLEX2* (Dolomanov *et al.*, 2009 ▸).

## supplementary crystallographic information

### Crystal data

**Table:**

[UO_2_(C_5_H_7_O_2_)_2_(H_2_O)]	*D*_x_ = 1.992 Mg m^−^^3^
*M**_r_* = 486.26	Mo *K*α radiation, λ = 0.71073 Å
Trigonal, *P*3*c*1	Cell parameters from 9890 reflections
*a* = 19.5774 (9) Å	θ = 3.2–25.4°
*c* = 7.3264 (5) Å	µ = 10.03 mm^−^^1^
*V* = 2431.8 (3) Å^3^	*T* = 298 K
*Z* = 6	Needle, yellow
*F*(000) = 1344	0.30 × 0.15 × 0.05 mm

### Data collection

**Table:**

Bruker D8 Quest PHOTON II diffractometer	1426 reflections with *I* > 2σ(*I*)
ω–scan	*R*_int_ = 0.027
Absorption correction: multi-scan (SADABS; Krause *et al.*, 2015)	θ_max_ = 25.4°, θ_min_ = 3.5°
*T*_min_ = 0.457, *T*_max_ = 0.745	*h* = −23→23
26571 measured reflections	*k* = −23→23
1493 independent reflections	*l* = −8→8

### Refinement

**Table:**

Refinement on *F*^2^	Primary atom site location: dual
Least-squares matrix: full	Secondary atom site location: difference Fourier map
*R*[*F*^2^ > 2σ(*F*^2^)] = 0.016	Hydrogen site location: mixed
*wR*(*F*^2^) = 0.042	H atoms treated by a mixture of independent and constrained refinement
*S* = 1.13	*w* = 1/[σ^2^(*F*_o_^2^) + (0.0203*P*)^2^ + 3.5627*P*] where *P* = (*F*_o_^2^ + 2*F*_c_^2^)/3
1493 reflections	(Δ/σ)_max_ < 0.001
89 parameters	Δρ_max_ = 0.51 e Å^−^^3^
0 restraints	Δρ_min_ = −1.06 e Å^−^^3^

### Special details

**Table:**

Geometry. All esds (except the esd in the dihedral angle between two l.s. planes) are estimated using the full covariance matrix. The cell esds are taken into account individually in the estimation of esds in distances, angles and torsion angles; correlations between esds in cell parameters are only used when they are defined by crystal symmetry. An approximate (isotropic) treatment of cell esds is used for estimating esds involving l.s. planes.
Refinement. Refinement of *F*^2^ against ALL reflections. The weighted *R*-factor *wR* and goodness of fit *S* are based on *F*^2^, conventional *R*-factors *R* are based on *F*, with *F* set to zero for negative *F*^2^. The threshold expression of *F*^2^ > 2σ(*F*^2^) is used only for calculating *R*-factors(gt) *etc*. and is not relevant to the choice of reflections for refinement. *R*-factors based on *F*^2^ are statistically about twice as large as those based on *F*, and *R*- factors based on ALL data will be even larger.A suitable crystal of [UO2(acac)2(H2O)] was selected and mounted on a Bruker D8 Quest diffractometer. The crystal was kept at 298.0?K during data collection. Using Olex2 (Dolomanov, 2009) the structure was solved with the SHELXT (Sheldrick 2015) structure solution program using Intrinsic Phasing and refined with the SHELXL (Sheldrick 2015) refinement package using Least Squares minimisation.

### Fractional atomic coordinates and isotropic or equivalent isotropic displacement parameters (Å^2^)

**Table:**

	*x*	*y*	*z*	*U*_iso_*/*U*_eq_	
U1	0.38611 (2)	0.000000	0.750000	0.03158 (8)	
O4	0.5083 (2)	0.000000	0.750000	0.0415 (8)	
O1	0.45776 (16)	0.08101 (15)	0.4979 (3)	0.0442 (6)	
O2	0.31937 (16)	0.06227 (16)	0.6287 (4)	0.0525 (7)	
O3	0.42991 (16)	0.08004 (14)	0.9047 (3)	0.0453 (6)	
C1	0.4697 (2)	0.1489 (2)	0.4498 (5)	0.0437 (9)	
C2	0.4199 (3)	0.1769 (2)	0.4902 (6)	0.0524 (10)	
H2	0.436802	0.229520	0.464721	0.063*	
C3	0.3450 (3)	0.1311 (3)	0.5675 (5)	0.0503 (9)	
C4	0.5432 (3)	0.1979 (3)	0.3397 (7)	0.0674 (13)	
H4A	0.588238	0.204246	0.406406	0.101*	
H4B	0.548844	0.248675	0.316338	0.101*	
H4C	0.539460	0.171870	0.225884	0.101*	
C5	0.2896 (3)	0.1632 (4)	0.5750 (7)	0.0742 (15)	
H5A	0.249096	0.134214	0.663397	0.111*	
H5B	0.266109	0.158127	0.457143	0.111*	
H5C	0.318349	0.217860	0.609141	0.111*	
H4	0.525 (3)	−0.018 (3)	0.694 (7)	0.073 (17)*	

### Atomic displacement parameters (Å^2^)

**Table:**

	*U*^11^	*U*^22^	*U*^33^	*U*^12^	*U*^13^	*U*^23^
U1	0.03345 (9)	0.02596 (10)	0.03283 (11)	0.01298 (5)	0.00110 (3)	0.00219 (6)
O4	0.0423 (15)	0.044 (2)	0.0383 (19)	0.0222 (10)	−0.0026 (8)	−0.0052 (16)
O1	0.0552 (16)	0.0402 (14)	0.0435 (13)	0.0286 (12)	0.0131 (12)	0.0121 (11)
O2	0.0516 (16)	0.0580 (17)	0.0576 (18)	0.0345 (15)	0.0090 (13)	0.0207 (13)
O3	0.0560 (16)	0.0355 (13)	0.0447 (14)	0.0231 (12)	−0.0011 (12)	−0.0068 (11)
C1	0.054 (2)	0.0369 (19)	0.0394 (19)	0.0223 (18)	0.0035 (16)	0.0077 (15)
C2	0.068 (3)	0.041 (2)	0.054 (2)	0.032 (2)	0.011 (2)	0.0157 (18)
C3	0.071 (3)	0.061 (3)	0.0364 (19)	0.046 (2)	0.0031 (18)	0.0109 (18)
C4	0.062 (3)	0.054 (3)	0.082 (3)	0.025 (2)	0.022 (2)	0.027 (2)
C5	0.097 (4)	0.101 (4)	0.065 (3)	0.080 (3)	0.024 (3)	0.031 (3)

### Geometric parameters (Å, º)

**Table:**

U1—O4	2.392 (4)	C1—C2	1.369 (6)
U1—O1	2.381 (2)	C1—C4	1.504 (5)
U1—O1^i^	2.381 (2)	C2—H2	0.9300
U1—O2	2.361 (3)	C2—C3	1.400 (6)
U1—O2^i^	2.361 (3)	C3—C5	1.502 (5)
U1—O3	1.769 (2)	C4—H4A	0.9600
U1—O3^i^	1.769 (2)	C4—H4B	0.9600
O4—H4	0.71 (4)	C4—H4C	0.9600
O4—H4^i^	0.71 (4)	C5—H5A	0.9600
O1—C1	1.279 (4)	C5—H5B	0.9600
O2—C3	1.261 (5)	C5—H5C	0.9600
			
O1^i^—U1—O4	75.17 (6)	C1—O1—U1	130.3 (2)
O1—U1—O4	75.17 (6)	C3—O2—U1	131.1 (3)
O1^i^—U1—O1	150.33 (12)	O1—C1—C2	124.0 (3)
O2—U1—O4	144.24 (7)	O1—C1—C4	115.4 (4)
O2^i^—U1—O4	144.24 (6)	C2—C1—C4	120.5 (3)
O2^i^—U1—O1	139.25 (9)	C1—C2—H2	118.0
O2—U1—O1^i^	139.25 (9)	C1—C2—C3	124.0 (4)
O2^i^—U1—O1^i^	70.00 (9)	C3—C2—H2	118.0
O2—U1—O1	70.00 (9)	O2—C3—C2	124.0 (4)
O2^i^—U1—O2	71.52 (13)	O2—C3—C5	116.7 (4)
O3—U1—O4	87.61 (9)	C2—C3—C5	119.3 (4)
O3^i^—U1—O4	87.61 (9)	C1—C4—H4A	109.5
O3^i^—U1—O1^i^	92.50 (11)	C1—C4—H4B	109.5
O3—U1—O1^i^	86.27 (10)	C1—C4—H4C	109.5
O3—U1—O1	92.50 (11)	H4A—C4—H4B	109.5
O3^i^—U1—O1	86.27 (10)	H4A—C4—H4C	109.5
O3—U1—O2^i^	97.81 (12)	H4B—C4—H4C	109.5
O3—U1—O2	86.10 (11)	C3—C5—H5A	109.5
O3^i^—U1—O2	97.81 (12)	C3—C5—H5B	109.5
O3^i^—U1—O2^i^	86.10 (11)	C3—C5—H5C	109.5
O3—U1—O3^i^	175.21 (17)	H5A—C5—H5B	109.5
U1—O4—H4^i^	134 (4)	H5A—C5—H5C	109.5
U1—O4—H4	134 (4)	H5B—C5—H5C	109.5
H4—O4—H4^i^	92 (8)		
			
U1—O1—C1—C2	−26.9 (6)	O1—C1—C2—C3	−8.8 (7)
U1—O1—C1—C4	154.2 (3)	C1—C2—C3—O2	9.1 (7)
U1—O2—C3—C2	27.1 (6)	C1—C2—C3—C5	−169.0 (4)
U1—O2—C3—C5	−154.8 (3)	C4—C1—C2—C3	170.0 (4)

Symmetry code: (i) *x*−*y*, −*y*, −*z*+3/2.

### Hydrogen-bond geometry (Å, º)

**Table:**

*D*—H···*A*	*D*—H	H···*A*	*D*···*A*	*D*—H···*A*
O4—H4···O1^ii^	0.72 (6)	2.01 (5)	2.704 (3)	164 (7)

Symmetry code: (ii) −*x*+1, −*y*, −*z*+1.
